# A Novel Murine Cytomegalovirus Vaccine Vector Protects against *Mycobacterium tuberculosis*

**DOI:** 10.4049/jimmunol.1302523

**Published:** 2014-07-28

**Authors:** Peter C. L. Beverley, Zsolt Ruzsics, Ariann Hey, Claire Hutchings, Simone Boos, Beatrice Bolinger, Emanuele Marchi, Geraldine O'Hara, Paul Klenerman, Ulrich H. Koszinowski, Elma Z. Tchilian

**Affiliations:** * Nuffield Department of Medicine, University of Oxford, Oxford OX1 3SY, United Kingdom; and; † Max von Pettenkofer Institute, Ludwig Maximilians University, D-80336 Munich, Germany

## Abstract

Tuberculosis remains a global health problem so that a more effective vaccine than bacillus Calmette–Guérin is urgently needed. Cytomegaloviruses persist lifelong in vivo and induce powerful immune and increasing (“inflationary”) responses, making them attractive vaccine vectors. We have used an m1–m16-deleted recombinant murine CMV (MCMV) expressing *Mycobacterium tuberculosis* Ag 85A to show that infection of mice with this recombinant significantly reduces the mycobacterial load after challenge with *M. tuberculosis*, whereas control empty virus has a lesser effect. Both viruses induce immune responses to H-2^d^–restricted epitopes of MCMV pp89 and M18 Ags characteristic of infection with other MCMVs. A low frequency of 85A-specific memory cells could be revealed by in vivo or in vitro boosting or after challenge with *M. tuberculosis.* Kinetic analysis of *M. tuberculosis* growth in the lungs of CMV-infected mice shows early inhibition of *M. tuberculosis* growth abolished by treatment with NK-depleting anti–asialo ganglio-*N*-tetraosylceramide Ab. Microarray analysis of the lungs of naive and CMV-infected mice shows increased IL-21 mRNA in infected mice, whereas in vitro NK assays indicate increased levels of NK activity. These data indicate that activation of NK cells by MCMV provides early nonspecific protection against *M. tuberculosis,* potentiated by a weak 85A-specific T cell response, and they reinforce the view that the innate immune system plays an important role in both natural and vaccine-induced protection against *M. tuberculosis*.

## Introduction

Tuberculosis (TB) remains an important cause of morbidity and mortality worldwide, with 8.7 million new infections and 1.4 million deaths annually, and the difficulty of reducing this disease burden is compounded by the spread of drug-resistant organisms and of coinfection with HIV and TB. Furthermore, *Mycobacterium tuberculosis* has coexisted with humans for 70,000 y, giving time for the organism to evolve many immune evasion strategies (1). Nevertheless, vaccination is an attractive containment strategy for the TB epidemic, but the failure of the recombinant modified vaccinia Ankara virus booster vaccine, MVA85A, in a recent phase IIB trial and the relatively poor protection generated by many candidate vaccines in animal models indicate the difficulties (2–4).

Until now bacillus Calmette–Guérin (BCG) has been the only licensed vaccine for TB, and although vaccine efficacy varies geographically and protection wanes with age (5), BCG does protect against tuberculous meningitis (6) and furthermore provides nonspecific protection against other childhood infections (7). This is paralleled by the observation that immunization with BCG induces a state of “trained” immunity in macrophages, increasing the ability of these cells to respond to diverse pathogens in vitro (8). Thus, the efficacy of BCG may depend not only on induction of an appropriate adaptive response but also on its ability to stimulate the correct innate response, a property also demonstrated for at least one experimental TB subunit vaccine (9). Novel vaccine strategies for TB need to take the importance of innate responses into account.

CMVs are ubiquitous β-herpes viruses that establish life-long infection, associated with low-level persistence within the host. Virus is shed from epithelial surfaces in body fluids (saliva, urine, breast milk, and genital secretions) and transmission is by close contact and exposure to these secretions. Infection is acquired most commonly in infancy or adolescence and is often asymptomatic, although CMVs can cause life-threatening infection prenatally and in immunosuppressed patients (10, 11), and unusually, although the viruses are highly immunogenic, CMVs can reinfect already infected patients.

CD8 T cell responses to some CMV Ags exhibit “memory inflation,” so that instead of an initial response postinfection followed by a rapid contraction and maintenance of a smaller memory population, the responding population increases slowly over time or is maintained at a high level. Importantly, the responding cells have an activated effector or effector memory phenotype, which facilitates entry of the cells into nonlymphoid tissues such as the lung and genital mucosa (12). These properties have prompted exploration of CMVs as vaccine vectors against SIV, Ebola virus, and HSV (13–15). Unusually, one rhesus monkey CMV-based vaccine vector induces CD8 T cells restricted by MHC class II, as well as a broad repertoire of T cells specific for many epitopes of the vaccine Ags, properties that may account for its efficacy in protecting against SIV challenge in macaques (16).

The ability to make rCMVs has been facilitated by bacterial artificial chromosome (BAC) technology, and vaccine vectors have been constructed expressing Ags under several different promoters. Furthermore, BAC technology has made it simple to investigate the function of CMV genes and study the effect of removing those known to interfere with immune function to improve the protective efficacy of CMV-based vaccines (17–19).

In the present study, we investigate to our knowledge for the first time the ability of a novel recombinant murine CMV (MCMV) vector, in which the first 16 genes of the virus, including some that interfere with expression of MHC class I having been deleted (17–20), to induce protective immunity against a bacterial pathogen *M. tuberculosis*. Although MVA85A did not show efficacy in humans (2), recombinant adenoviruses expressing *M. tuberculosis* Ag 85A have been shown to protect in mice (21), guinea pigs (22), and cattle (23), so this well-characterized *M. tuberculosis* mycolyl transferase was used as a vaccine Ag in our rMCMV, expressed under the control of the human CMV promoter.

We show that this recombinant virus, MCMV85A, induces strong protection against challenge with *M. tuberculosis*, whereas the control virus, eMCMV, has a weaker, nonspecific effect. We examine the mechanisms of protection.

## Materials and Methods

### Viruses and vector construction

The rMCMV constructs were generated on the Δm1-16-FRT backbone (24), which is derived from pSM3fr (25) and therefore is m129 negative (26). This was modified by insertion of an IRES-controlled mCherry cassette at the 3′ untranslated region of the gene M48.5, allowing fluorescent detection of viral replication in tissue culture (27). The MCMV85A-BAC was generated by insertion pO6-A5-CMV-TB85A into the Δm1-16-FRT-BAC by Flp-mediated recombination as described (28). pO6-A5-CMV–*M. tuberculosis* 85A was generated by inserting a PCR-generated fragment of a codon-optimized synthetic 85A open reading frame (ORF) fused to the signal sequence of the human tissue plasminogen activator upstream sequence for better expression in eukaryotic cells (29), into pO6-A5-CMV-gfp (Sirion Biotech, Martinsried, Germany) using NheI/NotI digestion to replace the gfp coding sequence. The TB85 ORF was amplified using primers NTPA forward (5′-TCCGCTAGCATGGATGCAATGCAATGAAGAGAGGGCT-3′) and 85 reverse (5′-GATCGCGGCCGCGGATCCTAGGCGCCCTGGGGCGCGGGC-3′); the restriction sites used are underlined. The eMCMV-BAC was generated by insertion of pO6-A5-CMV into Δm1-16-FRT-BAC. pO6-A5-CMV was generated from pO6-A5-CMV-gfp by removal of the gfp coding sequence with NheI/NotI. The viruses MCMV85A and eMCMV were reconstituted from the respective BACs after transfection of mouse embryo fibroblasts as described (28).

Adenovirus expressing Ag 85A (Ad85A) is a recombinant human adenovirus type 5 expressing *M. tuberculosis* Ag85A. The virus was purified using Adenopure columns (Puresyn, Malvern, PA) and used as a positive control vaccine (30).

### Infectious virus propagation and quantitation

MCMV85A and eMCMV were propagated and titrated in NIH 3T3 cells (European Cell Culture Collection, Porton Down, U.K.). For propagation, monolayers of NIH 3T3 cells grown in DMEM (Life Technologies, Paisley, U.K.) were infected with eMCMV or MCMV85A and incubated for 4 d. Supernatants were harvested and stored at −80°C.

To detect expression of 85A in vitro, RNA was isolated from 3T3 cells infected with MCMV85A 48 h earlier at a multiplicity of infection of 100:1. Cells were trypsinized, centrifuged, and the pellet was resuspended in 1 ml TRIzol reagent (Life Technologies). RNA was extracted using the RNeasy kit (Qiagen, Hilden, Germany), DNAse treated (DNA-free kit; Ambion/Life Technologies, Carlsbad, CA), and reverse transcribed using a high-capacity cDNA reverse transcription kit (Applied Biosystems/Life Technologies, Foster City, CA) according to the manufacturers’ instructions. PCR was performed using forward (5′-CAGGACGACTTCAGCGGC-3′) and reverse (5′-TCGCCAGCGTCAGCGCCG-3′) primers amplifying a 277-bp product of the 85A sequence (nt 134–411).

Virus titers in organs of eMCMV- or MCMV85A-infected mice were determined by plaque assay on NIH 3T3 cells as described (31).

### MCMV infection and immunization of mice

All animal work was carried out in accordance with the U.K. Animal (Scientific Procedures) Act 1986 and was approved by the Animal Use Ethical Committee of Oxford University.

Six- to 8-wk-old female BALB/c mice (Harlan Orlac, Blackthorn, U.K.) were infected i.p. or i.v. with 2 × 10^6^ PFU eMCMV or MCMV85A. In some experiments mice were immunized intranasally (i.n.) with 2 × 10^9^ virus particles Ad85A or s.c. with 2 × 10^5^ CFU BCG as described previously (30).

### Isolation of lymphocytes from lungs, spleen, and livers

Lungs were perfused with PBS, cut into pieces, and digested with 0.7 mg/ml collagenase type I (Life Technologies) and 30 μg/ml DNase I (Sigma-Aldrich, Dorset, U.K.) for 45 min at 37°C. Digested fragments were crushed through a cell strainer using a syringe plunger, washed, RBCs were removed with lysis buffer (Qiagen) and, after centrifugation, resuspended in HEPES-buffered RPMI 1640 containing 10% FCS and 1% penicillin-streptomycin. Spleens were passed through a cell strainer using a syringe plunger, RBCs were removed with lysis buffer (Qiagen), and the cells were washed. Livers were passed through a cell strainer, the cells washed, and lymphocytes were isolated by gradient centrifugation using Percoll (GE Healthcare, Uppsala, Sweden).

### In vitro restimulation of T cells

Splenic lymphocytes from MCMV85A-infected mice were allowed to adhere to plastic for 2 h at 37°C in HEPES-buffered RPMI 1640 containing 10% FCS and 1% penicillin-streptomycin. The nonadherent cells were recovered and mixed 1:1 with naive spleen cells. The mixture was adjusted to 10^7^ cells/ml and 1 ml was added to 24-well tissue culture plates. Medium or medium containing a pool of 58 peptides (15-mer each) overlapping by 10 aa covering the entire 85A sequence was added in 1 ml to give a final concentration for all peptides of 2 μg/ml. After 4 d culture at 37°C, 1 ml medium was removed and replaced by medium containing 2 ng/ml recombinant human IL-2 (Life Technologies). After a further 3 d the nonadherent cells were recovered from each well, washed, and returned to the same well for overnight culture. The following day the proportion of Ag-specific cytokine-producing cells in the rested cells was determined by flow cytometry after restimulation with 85A peptides, as described below.

### NK cell assay

YAC-1 NK-sensitive target cells (32) were labeled with CFSE (Invitrogen) as previously described (30). To 10,000 labeled target cells spleen cells were added at different attacker/target ratios in round-bottom 96-well plates and the plates were centrifuged for 3 min at 1500 rpm before incubation for 16 h at 37°C. The plates were centrifuged again and the supernatant was removed, the cells were resuspended by vortexing, and 50 μl fixable Live/Dead cell stain was added (Molecular Probes). After 20 min at 4°C, 100 μl FACS buffer was added to each well, the plate was centrifuged, the supernatant was removed, cells were resuspended, and 200 μl FACS buffer was added. An aliquot of 100 μl was removed from each well and added to an equal volume of medium containing a known number (∼10^4^) of fluorescent microbeads. The ratio of viable target cells to beads was determined by flow cytometry and percentage cytotoxicity calculated by the formula: % cytotoxicity = 1 − [(ratio of viable target cells/beads at each attacker/target ratio)/(ratio of viable cells alone/beads)] × 100.

### Flow cytometry

Lymphocytes were stimulated in RPMI 1640 containing 10% FCS and 1% penicillin-streptomycin for 6 h with the H-2^d^–restricted MCMV CD8 peptide epitopes (SGPSRGRII) from the MCMV M18 (33) or (YPHFMPTNL) from the pp89 Ag. For Ag 85A, cells were stimulated either with the pool of 58 peptides (15-mer each) or with three H-2^d^–restricted peptides encoding the dominant CD4 85A_99–118aa_ (TFLTSELPGWLQANRHVKPT) or CD8 85A_70–78aa_ (MPVGGQSSF) and 85A_145–152aa_ (YAGAMSGL) epitopes (30, 34, 35) (peptides were synthesized at the Weatherall Institute of Molecular Medicine, Oxford, U.K.). Each peptide was used at 2 μg/ml. After 1 h at 37°C, GolgiPlug (BD Biosciences, Oxford, U.K.) was added according to the manufacturer’s instructions.

Cells were washed and incubated with anti-mouse CD16/CD32 (clone 93) to block Fc binding (eBioscience, Hatfield, U.K.). Subsequently, the cells were stained for CD4 (RM4-5), CD8 (53-6.7) (BD Biosciences), fixable Live/Dead cell stain (Molecular Probes), IFN-γ (XMG1.2), IL-2 (JES6-5H4), TNF-α (MP6-XT22), CD127 (A7R34), CD62L (MEL-14), KLRG1 (2F1), and CD27 (LG.7F9) (all eBioscience) using the BD Cytofix/Cytoperm kit according to the manufacturer’s instructions. Cells were run on an LSR II (BD Biosciences) and analyzed using FlowJo software (Tree Star, Ashland, OR). Background responses of unstimulated cells were subtracted from the stimulated responses.

### Infection with *M. tuberculosis* and determination of mycobacterial load

MCMV-infected, immunized, or naive groups of four to seven mice were anesthetized with isoflurane/oxygen and infected i.n. with *M. tuberculosis* (Erdman strain) in 40 μl PBS equally divided between both nostrils. Lung CFU were enumerated 24 h after challenge to determine the number of organisms deposited (∼200 CFU). Mice were sacrificed at indicated times, the lungs and spleen were homogenized, and mycobacterial load was determined by plating 10-fold serial dilutions of tissue homogenates on Middlebrook 7H11 agar plates (E&O Laboratories, Bonnybridge, U.K.). CFU were counted after 3–4 wk of incubation at 37°C in 5% CO_2_. We have previously shown that i.n. challenge gives identical results to aerosol challenge with a Henderson apparatus (30).

### NK cell depletion

NK cells of naive, eMCMV-infected, or MCMV85A-infected mice were depleted by two i.p. injections of 50 μl anti–asialo ganglio-*N*-tetraosylceramide (ASGM1) Ab (Alpha Laboratories) or 100 μg normal rabbit Ig (Sigma-Aldrich) as a control 4 d before and on the day of *M. tuberculosis* challenge.

NK depletion was assessed by staining of lung and spleen cells for NKp46 (29A1.4; eBioscience) and DX5 (BD Pharmingen) in addition to CD4, CD8, and Live/Dead marker. Nondepleted mice were used as control.

### Manipulation of IL-21 in vivo

Recombinant IL-21RFc or control human IgG1 (75 μg) (R&D Systems) was administered i.p. to eMCMV-infected mice 2 d before and on the day of challenge with *M. tuberculosis*. Mice were sacrificed 7 d later and *M. tuberculosis* CFU were enumerated. Recombinant mouse IL-21 (100 ng in PBS) (R&D Systems) or CCL22 (R&D Systems) as control was administered i.n. on the day of *M. tuberculosis* challenge, mice were sacrificed 7 d later, and *M. tuberculosis* CFU were enumerated.

### Microarrays

Gene expression analysis was performed on purified lung mononuclear cells of four uninfected and four eMCMV-infected mice 15 d postinfection using Agilent whole mouse genome oligonucleotide arrays (Miltenyi Biotec). The raw and normalized microarray data are downloadable from the Gene Expression Omnibus database (http://www.ncbi.nlm.nih.gov/geo/), accession number GSE57572.

Microarray data were preprocessed using the R language (http://cran.r-project.org/) and Bioconductor packages (http://www.bioconductor.org/). Array normalization was performed using the “vsn” algorithm and low signal features were filtered out from subsequent analysis. The Limma package was used to infer differentially expressed genes. After *p* value adjustment, 338 probes were found significant at *p* < 0.05.

### Statistical analysis

Data were analyzed by one-way ANOVA followed by a Tukey multiple comparison test.

## Results

### Generation of rMCMV expressing Ag 85A

The rMCMV constructs were generated by insertion into the Δm1-16-FRT backbone (24) of an IRES-controlled mCherry cassette at the 3′ untranslated region of the M48.5 gene (27). The MCMV85A-BAC was generated by Flp-mediated insertion of pO6-A5-CMV-TB85A, carrying a codon-optimized synthetic TB85A ORF under the control of the human CMV immediate early promoter (Supplemental Fig. 1A). The eMCMV-BAC was generated by insertion of the empty vector. MCMV85A and eMCMV were reconstituted from the respective BACs after -transfection of mouse embryo fibroblasts (28). The Δm1-16-FRT–based constructs are derived from pSM3fr (25) and are therefore MCK-2 (m129) negative (26). Additionally, the constructs lack genes m01–m16. This region includes two of the three MCMV genes, m04 and m06, which downregulate MHC class I–mediated Ag presentation, whereas m152 is intact. The replication of Δm1-16-FRT–derived constructs has been studied extensively in tissue culture where they replicate with wild-type kinetics (24, 27, 36).

Expression of the 85A gene was assessed by RT-PCR analysis of NIH 3T3 cells infected with eMCMV or MCMV85A. Only MCMV85A-infected 3T3 cells generate a PCR product consistent with the expression of 85A RNA in the cells (Supplemental Fig. 1B). To ensure that the rMCMVs had comparable growth characteristics, virus growth was assessed after in vivo infection in the salivary glands, liver, and spleens of mice at 6 d postinfection. Supplemental Fig. 1C shows that eMCMV and MCMV85A replicate comparably, suggesting that the insertion of 85A does not interfere with viral replication in vivo.

### MCMV85A protects against pulmonary challenge with *M. tuberculosis*

To determine the efficacy of eMCMV and MCMV85A in protection against *M. tuberculosis* challenge, BALB/c mice were infected i.p. with 2 × 10^6^ PFU eMCMV or MCMV85A and 4, 8, 12, or 24 wk later challenged with *M. tuberculosis*. Mice were also infected i.v. and challenged 4 wk later. Controls were either uninfected or immunized with Ad85A i.n., a highly protective subunit vaccine in this mouse strain, or BCG s.c. (21, 30). The mycobacterial load in the lungs and spleens was determined 5 wk after *M. tuberculosis* challenge. Although the i.v. route induced better protection at 4 wk after MCMV infection, because MCMV virus stocks produced in vitro are of low titer it is difficult to give the volumes required for high doses by the i.v. route, so the i.p. route was used for further analysis.

**FIGURE 1.**
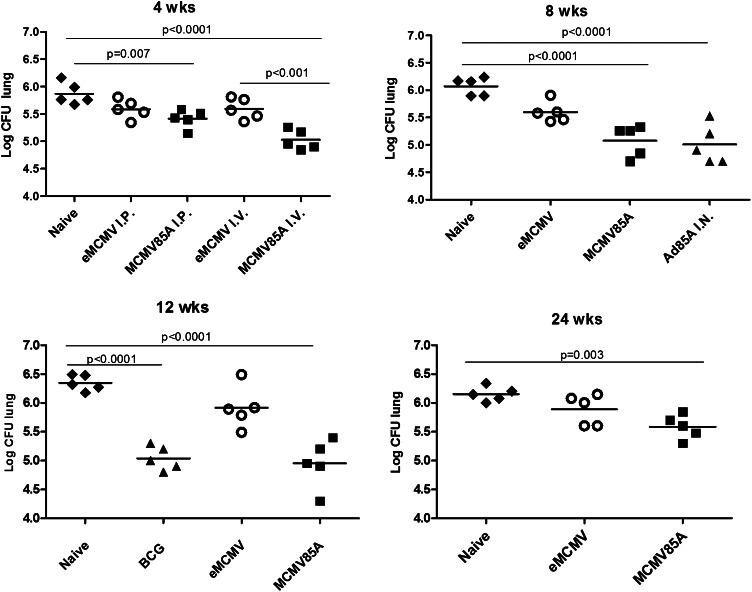
Protection against *M. tuberculosis* challenge. BALB/c mice were infected with 2 × 10^6^ PFU eMCMV or MCMV85A i.p. or i.v. and 4 wk later challenged with *M. tuberculosis.* Mice challenged at 8, 12, and 24 wk postinfection were immunized i.p. Controls were naive or immunized with 2 × 10^9^ virus particles Ad85A i.n. or 2 × 10^5^ CFU BCG s.c. Lung *M. tuberculosis* CFU were enumerated 5 wk after challenge. Symbols show individual mice, and the horizontal line indicates the mean. Data were analyzed by a one-way ANOVA with a Tukey posttest.

Irrespective of the route of infection, at all times after MCMV85A infection, there is a significant reduction in mycobacterial load in the lungs compared with naive animals. At 4 wk there is significantly greater protection in MCMV85A i.v. infected than in eMCMV i.v. infected animals. At 8 wk the reduction in MCMV85A i.p. infected mice is comparable to that found in Ad85A i.n. immunized animals assayed simultaneously and at 12 wk to BCG. As in Ad85A-immunized mice (30), protection is maintained for at least 24 wk in MCMV85A-infected mice (Fig. 1). eMCMV-infected animals almost always show a trend toward a reduction in lung mycobacterial load, but this does not reach statistical significance in the experiments shown. Splenic CFU show similar trends to the lungs (not shown).

These results indicated that MCMV85A induces protection against *M. tuberculosis*, sustained for at least 24 wk and comparable to that obtained with the most effective subunit vaccine, Ad85A delivered i.n., or BCG, the “gold standard” TB vaccine.

### MCMV-specific immune responses

Because it is difficult to detect even wild-type MCMV beyond the first few weeks after infection in vivo in mice (37, 38), we studied the immune response to well-characterized H-2^d^–restricted MCMV antigenic epitopes to determine whether the attenuated eMCMV or MCMV85A viruses persist in vivo and generate immune responses characteristic of other MCMVs.

**FIGURE 2.**
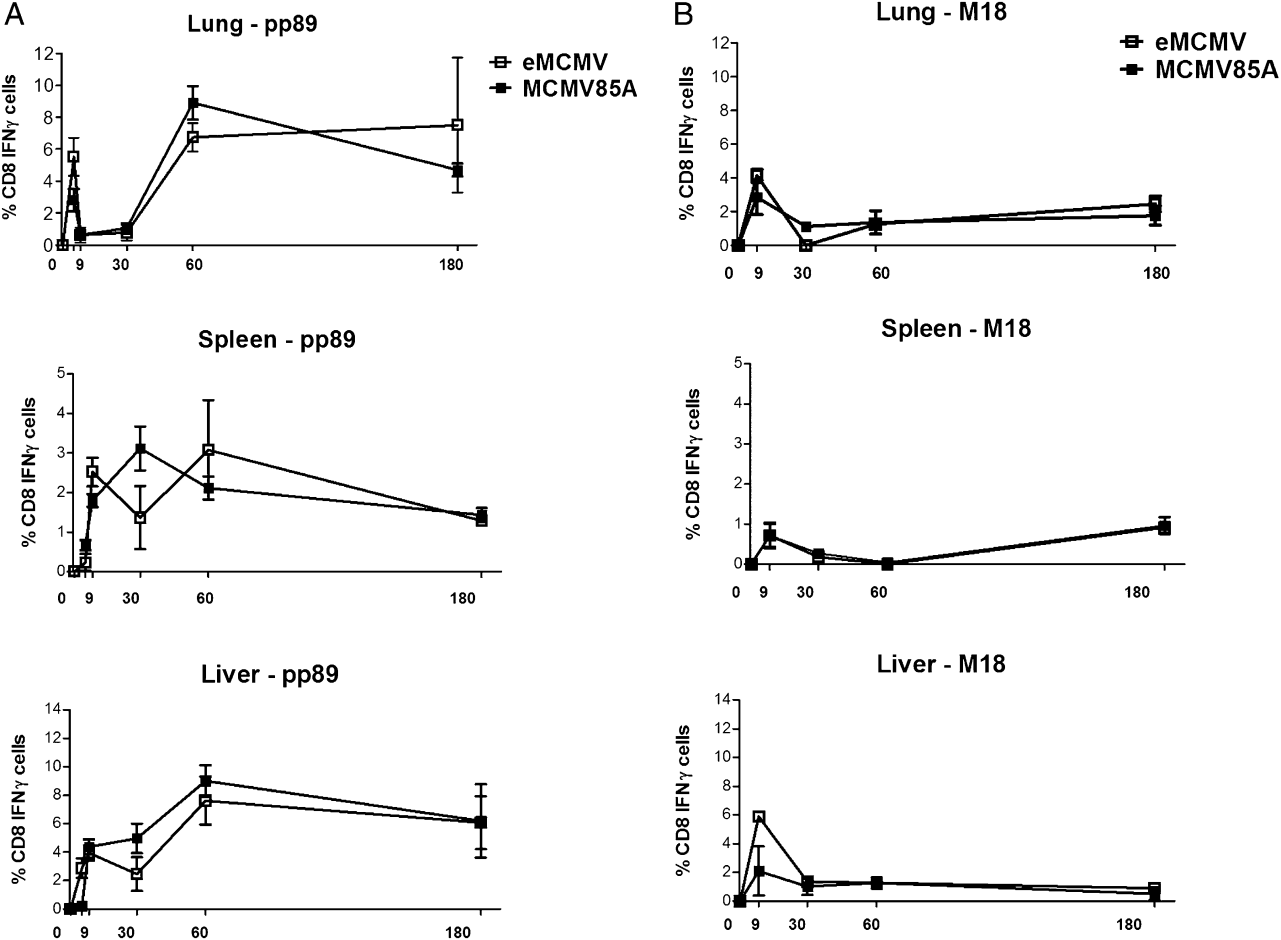
Kinetics of pp89 and M18 responses. BALB/c mice were infected with 2 × 10^6^ PFU eMCMV or MCMV85A i.p. Lung, liver, and spleen cells were isolated at the indicated times and after stimulation for 6 h with pp89 (**A**) or M18 (**B**) peptides, and the proportion of CD8 IFN-γ–producing cells was determined by surface and intracellular immunofluorescence staining and flow cytometric analysis. Results are expressed as the means ± SD of three to four mice per group.

The H2-L^d^–restricted CD8 T cell response to the immunodominant epitope (amino acid YPHFMPTNL) from the IE1 protein m123/phosphoprotein 89 (pp89) (39) shows an initial peak at day 9 after MCMV infection followed by a decline. However, after day 30, pp89-specific T cell frequencies rise again so that at day 180 postinfection ∼5–8% of the CD8 cells in the lung, ∼1% in the spleen, and ∼6% in the liver are pp89 specific (Fig. 2A). In contrast, the H2-D^d^–restricted CD8 response to an epitope (amino acid SGPSRGRII) from the M18 early gene (33) peaks at day 9 followed by a decline, with frequencies at day 180 of M18-specific cells remaining detectable at ∼1.7% in the lung, ∼1% in the spleen, and 0.5% in the liver (Fig. 2B). There are no significant differences between the frequencies and kinetics of pp89- (Fig. 2A) or M18-specific (Fig. 2B) cells in eMCMV- versus MCMV85A-infected animals.

We also determined the differentiation and activation status of pp89- and M18-specific CD8 T cells, using CD127 and CD62L to define memory subpopulations (Supplemental Fig. 2) and increased KLRG1 and decreased CD27 to define the extent of activation (Supplemental Fig. 3) among the Ag-specific cells of MCMV85A- or eMCMV-infected mice. At 60 d postinfection with MCMV85A, in the lungs ∼61% of pp89-specific cells are effectors compared with 28% of M18-specific cells, whereas 57% of M18-specific and 37% of pp89-specific cells are effector memory. Similar trends are seen for eMCMV at day 60 and for both viruses at day 180 (data not shown). At both time points, pp89-specific cells show increased KLRG1 and decreased CD27 Ag expression (40) compared with M18-specific cells, and also lung and liver Ag-specific cells are more activated than splenic cells (Supplemental Fig. 3).

The kinetics of the pp89 and M18 responses confirm their inflationary and noninflationary nature, respectively. In agreement with previous studies, pp89-specific cells exhibit a more activated phenotype than do M18-specific cells, especially in the lungs and liver. Overall, these data indicated that the two vectors persist in vivo, behave similarly to previously described MCMVs, and induce similar responses to MCMV Ags at the time points tested (39, 41, 42).

### Ag 85A–specific responses

We next assessed the response to Ag 85A in the MCMV85A-infected animals. A pool of 58 peptides (15-mer each), covering the whole sequence of 85A protein, was used to stimulate lung, liver, and spleen cells from MCMV85A- or eMCMV-infected mice for 6 h in vitro, and intracellular staining for IFN-γ, TNF-α, and IL-2 was performed but we could not demonstrate convincingly a reproducible, continuing, and statistically significant 85A-specific response at 3, 6, 9, 30, 60, or 180 d postinfection. Neither are cytokine-producing cells from MCMV85A-infected mice demonstrable when individual known dominant H-2^d^–restricted peptide epitopes of Ag 85A (I-E^d^ CD4_99–118_, L^d^ CD8_70–78_, and K^d^ CD8_145-152_) (34, 35) are used for stimulation. Furthermore, no T cell response to 85A can be detected by ELISPOT, nor is 85A-specific Ab detectable by ELISA in sera from MCMV85-infected animals (data not shown).

**FIGURE 3.**
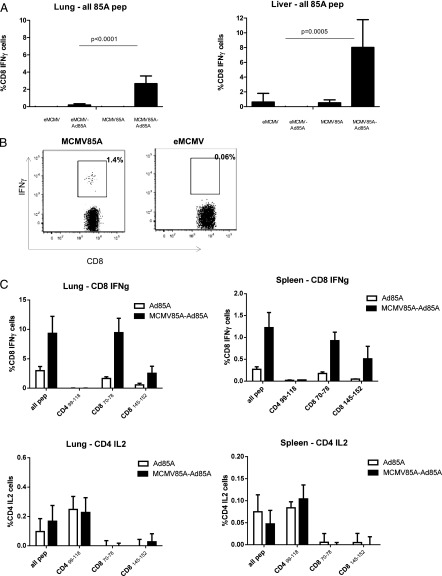
Ag 85A–specific immune responses. (**A**) Mice were infected with eMCMV or MCMV85A and 4 wk later boosted with 2 × 10^9^ virus particles Ad85A i.m. Six days later, lung, spleen, and liver cells were isolated and stimulated for 6 h with pooled 85A peptides. (**B**) Spleen cells from eMCMV- or MCMV85A-infected mice were stimulated in vitro with a pool of 15-mer peptides covering the sequence of Ag 85A, expanded with IL-2, rested, and restimulated with 85A peptides before assaying by intracellular staining and flow cytometry for Ag-specific IFN-γ–producing cells. (**C**) Lung or spleen cells from MCMV85A-infected mice boosted with Ad85A were stimulated with individual 85A peptides covering the dominant H-2^d^ CD4 and CD8 epitopes. The frequencies of IFN-γ– or IL-2–producing cells were determined by flow cytometry on CD8- and CD4-gated cells. Results are expressed as the means ± SD of three or four mice per group, representative of two independent experiments. Data were analyzed by a one-way ANOVA with a Tukey posttest.

However, because the MCMV85A animals were better protected than eMCMV-infected mice, we reasoned that there must be an 85A-specific response and that it should be possible to reveal this by boosting in vivo or in vitro. Therefore, 4 wk after infection with either eMCMV or MCMV85A, we boosted the animals with Ad85A and examined the immune response 6 d later. At this early time after immunization, eMCMV-infected mice make a very small response to Ag 85A, but the response in MCMV85A-infected mice is much greater (Fig. 3A). Similarly, when spleen cells from MCMV85A are stimulated with 85 peptides in vitro in the presence of uninfected APCs and expanded in IL-2, a population of Ag-specific CD8 T cells can be detected (Fig. 3B).

In BALB/c mice after Ad85A immunization most of the CD8 responding cells react to the dominant 85A_70–78_ epitope and a smaller proportion to the 85A_145–152_ epitope. There is also a small but detectable CD4 response to the 85A_99–118_ epitope (40). In MCMV85A-infected mice boosted with Ad85A, responses to the CD8 dominant and subdominant epitopes are seen whereas the CD4 response is unchanged (Fig. 3C). In vitro–boosted and IL-2–expanded cells from MCMV85A-immunized animals show similar specificity (data not shown). Data on CD8 IFN-γ and CD4 IL-2–producing cells are shown, as these provide the most sensitive detection of the two subsets.

Overall, these results show that MCMV85A-infected animals have a low frequency of 85A-specific memory CD8 and CD4 cells, which can be revealed by boosting with Ad85A in vivo or with 85A peptides in vitro.

### Mechanism of protection against *M. tuberculosis*

**FIGURE 4.**
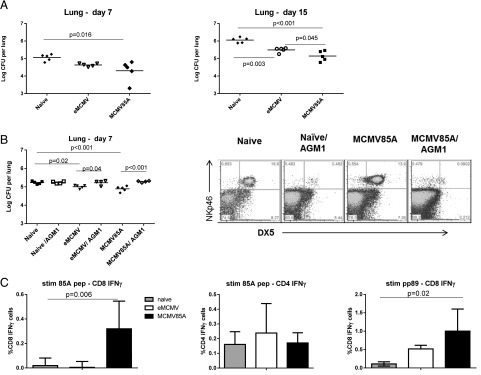
Mechanisms of *M. tuberculosis* protection. (**A**) Mice were infected with 2 × 10^6^ PFU eMCMV or MCMV85A i.p. with uninfected (naive) mice as controls. Five weeks postinfection all the mice were challenged with *M. tuberculosis* and sacrificed at days 7 and 15 for enumeration of lung CFU. (**B**) Five weeks postinfection with eMCMV or MCMV85A, infected and control uninfected (naive) mice were challenged with *M. tuberculosis* i.n. and after 1 wk sacrificed for enumeration of lung *M. tuberculosis* CFU. The day before and on the day of *M. tuberculosis* challenge, mice were given anti-ASGM1 Ab i.p. Flow cytometry panels show depletion by anti-ASGM1 Ab as assessed by DX5 and NKp46 staining of lung cells. Representative CFU data from one of two experiments with four to five mice per group are shown. (**C**) Mice were infected and challenged as in (A). Lung cells were isolated 14 d after the *M. tuberculosis* challenge and stimulated for 6 h with pooled 85A peptides or pp89. The frequencies of IFN-γ–producing cells were determined by flow cytometry on CD8- and CD4-gated cells. Results are expressed as the means ± SD of four mice per group. Data were analyzed by a one-way ANOVA with a Tukey posttest.

In most experiments eMCMV-infected animals show lower CFU than do naive animals, although this seldom reaches statistical significance (see Fig. 1). However, because the 85A-specific response is consistently low and yet protection by MCMV85A is equivalent to BCG and Ad85A i.n., two highly effective vaccines in mice, we wanted to better establish the mechanisms of protective immunity against *M. tuberculosis* in MCMV-infected mice. As we have previously shown that pulmonary local immune responses can inhibit *M. tuberculosis* growth very early after challenge, whereas systemic immune responses act only later (30), we first determined the kinetics of *M. tuberculosis* growth in the lungs of mice challenged with *M. tuberculosis* 5 wk postinfection with eMCMV or MCMV85A. Groups of mice were sacrificed 7 and 15 d later to determine the mycobacterial load in the lungs (Fig. 4A).

Both eMCMV and MCMV85A animals show a reduction in mycobacterial load at 7 and 15 d after challenge (Fig. 4A). The early reduction of *M. tuberculosis* load in eMCMV mice suggests that MCMV infection on its own induces early acting nonspecific protection against *M. tuberculosis,* potentiated by the specific response to 85A in MCMV85A mice. Because MCMV has been shown to activate NK cells, we used anti-ASGM1 Ab, given 4 d before and on the day of *M. tuberculosis* challenge, to deplete NK cells from naive, eMCMV-infected, or MCMV85A-infected mice. NK depletion alone does not affect *M. tuberculosis* CFU in the lungs of naive mice at 7 d after challenge, whereas it reverses the early protective effect in eMCMV- and MCMV85A-infected mice (Fig. 4B). Staining with DX5 and NKp46 Abs confirms the depletion of NK cells (Fig. 4B). In the experiment shown, a control Ab was not administered but a repeat experiment included control rabbit Ig. Identical results were obtained and control Ig had no effect. Although NK depletion abolishes protection in both eMCMV- and MCMV85A-infected mice, the latter show more effective control of *M. tuberculosis* at both 7 and 15 d (Fig. 4A), suggesting that an Ag-specific response potentiates the NK effect.

We therefore analyzed Ag-specific responses of lung cells of eMCMV- and MCMV85A-infected mice 15 d after *M. tuberculosis* challenge. Only the MCMV85A mice show an 85A-specific lung CD8 response (Fig. 4C), whereas a pp89-specific response is detected in both eMCMV- and MCMV85A-infected mice. As previously shown by others, all *M. tuberculosis*–challenged mice make a CD4 response to the pool of 85A peptides (34, 35).

Taken together, these data indicate that MCMV on its own has an early effect on mycobacterial growth, mediated mainly by NK cells, but the low frequency of 85A-specific memory cells present in MCMV85A-infected mice contributes to significantly improved protection.

**FIGURE 5.**
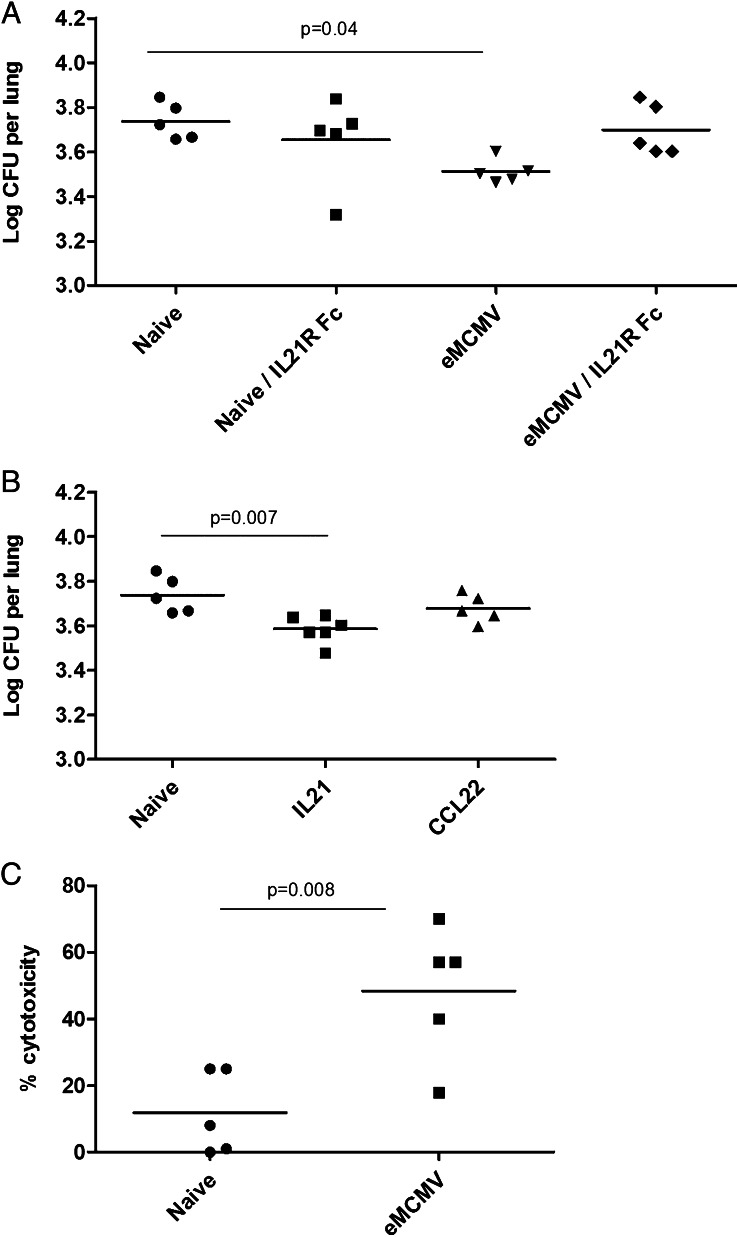
Effect of IL-21 on *M. tuberculosis* protection. (**A**) Mice infected with 2 × 10^6^ PFU eMCMV or uninfected controls were challenged with *M. tuberculosis*. Two days before and on the day of challenge, half of each group was treated with 75 μg IL-21RFc i.p. Mice were sacrificed 7 d later and *M. tuberculosis* CFU were enumerated. Human IgG1 was used as a control and had no effect (data not shown). (**B**) Naive mice were challenged with *M. tuberculosis.* On the days of challenge 100 ng recombinant mouse IL-21 or 100 ng CCL22 as control was administered i.n. Mice were sacrificed 7 d later and *M. tuberculosis* CFU were enumerated. (**C**) Spleen cells from five eMCMV and five uninfected mice were assayed on YAC-1 targets for NK activity. Data from 100:1 spleen cell to YAC-1 ratio are shown. Horizontal lines show the mean of each group. Data were analyzed by a one-way ANOVA with a Tukey posttest.

However, depletion of ASGM1-expressing cells is known to affect more than just NK cells (43, 44), so we sought further evidence for the role of these cells and carried out a microarray comparison of gene expression in the lungs of naive and eMCMV-infected animals (Supplemental Fig. 4). Microarray data were deposited in the Gene Expression Omnibus database (http://www.ncbi.nlm.nih.gov/geo/), accession number GSE57572. IL-21 expression, a cytokine known to be related to NK cell activity, is highly upregulated in eMCMV versus naive mice. Quantitative RT-PCR confirmed the upregulation of IL-21 message (IL-21 mRNA relative to hypoxanthine phosphoribosyltransferase in naive lungs of 0.00036 versus eMCMV lungs of 0.015, *p* = 0.02). We assessed the importance of IL-21 in two ways. First, we administered a soluble rIL-21RFc construct (45) systemically to MCMV-infected mice at the time of *M. tuberculosis* challenge to block IL-21 activity, and second, we tested whether rIL-21 administered to the lungs could induce early inhibition of *M. tuberculosis* growth. The results of these experiments are shown in Fig. 5. IL-21RFc blocks the early inhibitory effect on *M. tuberculosis* growth seen in eMCMV-infected mice (Fig. 5A), whereas IL-21 induces growth inhibition in normal mice (Fig. 5B), suggesting that IL-21 plays an important role in the nonspecific protective effect seen in eMCMV-infected mice, most likely through activation of NK cells (46, 47). In vitro assay of spleen cells from eMCMV mice confirms increased NK killing activity on YAC-1 cells compared with naive mice (Fig. 5C).

## Discussion

In many species, CMVs establish largely asymptomatic persistent infection, characterized by the accumulation of large numbers of virus-specific T cells (memory inflation) (39). These properties, as well as the fact that infection does not prevent reinfection, make rCMVs attractive vaccine vectors and they have been shown already to induce robust protection against SIV challenge in macaques and Ebola virus or HSV in mice (13–15). In this study, to our knowledge, we show for the first time that an rMCMV expressing *M. tuberculosis* Ag 85A confers protection against *M. tuberculosis* challenge in BALB/c mice. The reduction in mycobacterial load achieved by MCMV85A is comparable to that induced by Ad85A administered i.n. as well as the gold standard vaccine, parenteral BCG (21, 30, 48). Both MCMV85A and Ad85A induce protective immunity maintained for at least 24 wk (Fig. 1) (30).

Surprisingly, an immune response to Ag 85A was not detectable ex vivo in MCMV85A-infected mice and could be demonstrated only after in vivo boosting with Ad85A, following *M. tuberculosis* challenge or after in vitro stimulation in the presence of uninfected APCs, indicating that the MCMV85A-infected animals do have 85A-specific memory cells, but at a frequency not reliably detectable by intracellular cytokine staining. We considered several explanations for the low response to 85A. The first was that these vectors do not productively infect mice or persist in vivo. However, the vectors replicate in vitro similarly to wild-type MCMV (24, 27, 49) and they are capable of productive infection in vivo. Furthermore, they induce typical inflationary and noninflationary responses against pp89 and M18 epitopes. Because tissue culture grown MCMV is difficult to detect in vivo after the first few weeks of infection in immunocompetent mice (37) (so called “molecular latency”), virus can often only be revealed by transfer of tissues to SCID recipients. Therefore, persisting and inflating immune responses are commonly taken to indicate persistent infection (reviewed in Ref. 38), indicating that eMCMV and MCMV85A persist in vivo, as do similar attenuated MCMVs (49, 50). Another explanation for the low frequency of the 85A response might be antigenic competition between the dominant CD8 L^d^-restricted 85A_70–78_ epitope and the L^d^-restricted inflationary pp89 epitope. MCMV85A lacks the first 16 genes of the MCMV genome, including the m04 and m06 genes responsible for downregulation of MHC class I (17–20), so that presentation of MHC class I–restricted epitopes should not be inhibited in MCMV85A-infected cells because of the abundance of class I. However, m152, which is intact in this backbone, can downregulate D^d^ and L^d^ (19) and might potentiate competition, although both pp89 and 85A should be expressed with immediate early kinetics and should therefore be less sensitive to interference by the early gene m152 (38). Nevertheless, the possibility of antigenic competition for L^d^ is supported by data on the response to another L^d^-restricted epitope, M83/pp105, which is also maintained at a very low frequency, close to the limit of detection of intracellular cytokine staining (39). Thus, it may be that the pp89 epitope outcompetes other L^d^-restricted epitopes irrespective of the abundance of MHC class I, either because it has higher affinity for L^d^ or through other undetermined viral mechanisms.

The CD8 response in MCMV85A-infected animals, detected after boosting with Ad85A in vivo or with 85A peptides in vitro, is directed to MHC class I–restricted epitopes previously reported in animals immunized with Ad85A, 85A protein, or peptides (34, 35). There is no evidence for the unconventional MHC class II–restricted CD8 responses generated by recombinant rhesus monkey CMV vectors, but clearly these responses require expression of a particular set of CMV genes (16), which may not be present in MCMV85A.

Irrespective of the reason for the very low 85A-specific response in the MCMV85A-infected animals, the data illustrate an important principle, that it may not be necessary to induce a large Ag-specific population to obtain effective protection. This is in agreement with a recent study using an rMCMV virus containing an HSV-1 epitope, which showed that it is not necessary to induce an inflationary CD8 response for protection (13).

Our data indicate that in addition to the specificity and magnitude of the adaptive response, other factors may be critical for effective protection against *M. tuberculosis*. It is clear that MCMV itself has a nonspecific protective effect against *M. tuberculosis*, as has already been shown for *Listeria monocytogenes* for both MCMV (51) (P. Klenerman and G. O’Hara, personal communication) and murine gammaherpesvirus 68 (52). However, this effect, attributed to IFN-γ production and macrophage activation, has been reported to wane between 3 and 6 mo after virus infection (53), whereas there was still a trend toward a decreased mycobacterial load in eMCMV-infected mice at 24 wk (Fig. 1). It will be interesting to examine protection induced by both eMCMV and MCMV85A at even later time points after infection. Furthermore, although we have not excluded an effect of macrophage activation, this is largely dependent on MCK-2 in vivo, which is deficient in the Δm1-16-FRT backbone (54).

MCMV infection is known to efficiently activate NK cells, although the magnitude of this effect varies substantially depending on the host strain and virus variant (55, 56). Our microarray data indicate that the IL-21 message is upregulated in eMCMV-infected BALB/c mice, and we chose to investigate this gene further because it is known that a prominent effect of this highly pleiotropic member of the common γ-chain cytokine family is NK cell activation (57). Furthermore, pathway analysis showed that several genes related to IL-21 function are also highly upregulated (AICDA, RGS13, IL-10, MADCAM1). NK cell activation was confirmed by the increased NK cytotoxicity of cells from eMCMV mice. The importance of IL-21 and NK cells is supported by the in vivo experiments with IL-21RFc and IL-21, as well as the abolition of nonspecific protection by administration of anti-ASGM1 Ab. Interestingly, a plasmid expressing *M. tuberculosis* Ags 85A and ESAT-6 with IL-21 has been shown to give increased immune responses to the *M. tuberculosis* Ags, higher NK activity, and better protection against *M. tuberculosis*, compared with a plasmid without IL-21 (58).

In addition to the nonspecific effect, the 85A-specific cells induced by MCMV85A and recruited to or expanded in the lungs after *M. tuberculosis* challenge must play a role in protection (Fig. 4C) because MCMV85A consistently provides better protection than eMCMV, although whether the activated NK cells play a role in the activation of the Ag-specific CD8 cells remains to be determined.

As is the case with MCMV85A, BCG, the gold standard TB vaccine, is known to activate the innate immune system as well as induce an adaptive response (59). Furthermore, BCG induces a state of “trained immunity” in macrophages, persisting for several months and conferring increased nonspecific protection against other infections (8, 60), and recently cytokine responses of NK cells were identified as an effector mechanism induced by BCG (61).

Although the relative importance of innate and adaptive responses in BCG-induced protective immunity remains to be determined, in the case of MCMV85A, innate immunity appears to play an important role, although clearly potentiated by a weak adaptive response. However, even when a much more powerful and readily detectable adaptive response is induced, correct innate signaling remains crucial, as shown in experiments in which Ad85A and VSV85A were delivered to the lungs. Both vectors induce powerful, largely CD8, local immune responses, but whereas Ad85A is protective against *M. tuberculosis*, VSV85A is not, an outcome ascribed to a critical difference in the balance between IL-12 and type I IFN induced by the two vaccines (9).

To our knowledge, we have demonstrated specific anti–*M. tuberculosis* protection for the first time using novel MCMV vectors. Surprisingly, a small Ag-specific response may be highly effective in synergy with the correct innate activation in this case involving IL-21 and NK cells. There is clearly great scope for improving further the efficacy of CMV vectors (16, 51). New approaches are certainly required, because so far no vaccine against pathogens, for which T cells are the protective mechanism, has been successful, and the strategy of attempting to induce ever greater adaptive T cell responses has so far failed (2). Our data support the view that it is essential to induce correct innate as well as adaptive responses.

## Supplementary Material

Data Supplement
