# Supplementary material for: A Novel Murine Cytomegalovirus Vaccine Vector Protects against Mycobacterium tuberculosis
Source: J Immunol. 2014 Jul 28;193(5):2306–16. doi: 10.4049/jimmunol.1302523 (PMC4134927; doi:10.4049/jimmunol.1302523)
Supplement: Data Supplement [file supp_193_5_2306__index.html]

A Novel Murine Cytomegalovirus Vaccine Vector Protects against Mycobacterium tuberculosis — A Novel Murine Cytomegalovirus Vaccine Vector Protects against Mycobacterium tuberculosis — Data Supplement 

# A Novel Murine Cytomegalovirus Vaccine Vector Protects against *Mycobacterium tuberculosis*

## Data Supplement

**Files in this Data Supplement:**

- Supplemental Figures 1 (PDF)
