# Supplementary material for: A Novel Murine Cytomegalovirus Vaccine Vector Protects against Mycobacterium tuberculosis
Source: J Immunol. 2014 Jul 28;193(5):2306–16. doi: 10.4049/jimmunol.1302523 (PMC4134927; doi:10.4049/jimmunol.1302523)
Supplement: Data Supplement [file 1302523_JI_1302523_Supplemental_Figures_1.pdf]

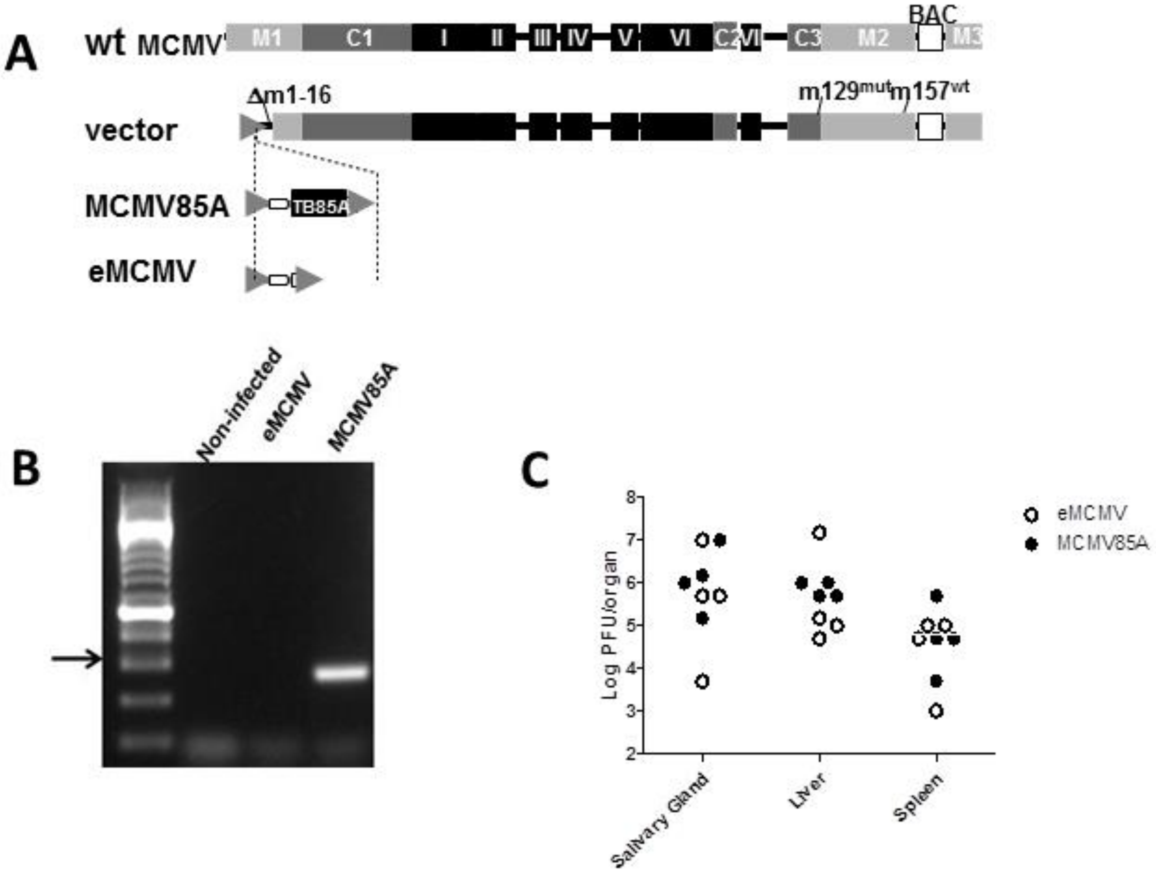


**Figure S1. Generation of eMCMV and MCMV85A**

(A) Schematic representation of the genetic engineering of the MCMV constructs. The upper schema shows the wt recombinant MCMV genome (wt MCMV): Gene blocks (I-VII, including genes M45-M116) that are conserved among all herpesviruses are located in the center of the genome (black boxes with Roman numerals). Within these gene blocks other genes are interspersed which show only a small degree of conservation. Additional gene clusters towards the ends of the genome are conserved in cytomegaloviruses (gray boxes, C1, C2 and C3; representing M23-M44, m106-m108 and m117-m131 respectively), and finally the terminal gene clusters (light gray boxes, M1 and M2 + M3 corresponding to m01-m22 and m132-m170) represent genes that are species specific. The BAC cassette (white box) is inserted into the right terminal species-specific gene cluster, splicing it artificially to M2 and M3, which remain functionally intact. In the vector backbone (Δm1-m16-FRT) used in this study and shown in the second schema, the terminal part of M1 including the genes m01 to m16 is deleted and an FRT site is inserted (gray arrowhead). Into this position expression cassettes are inserted by site-specific recombination. The expression cassette for MCMV85A (shown in the third schema) includes the *Mtb* 85A antigen (black box, TB85A) expressed under the control of the human CMV immediate early promoter and the BGH poly-adenylation signal. The control construct eMCMV depicted at the bottom contains only the empty transcription unit including the promoter and poly-adenylation signal. The vector backbone carries a mutant allele of m129 ($m129^{mut}$) and wild type m157 allele ($m157^{wt}$). (B) RT-PCR of 3T3 cells infected with MCMV85A. RNA was isolated from 3T3 cells infected with eMCMV or MCMV85A 48 hours after infection, reverse transcribed and amplified with 85A specific primers. The arrow indicates the 300bp marker. A strong 277bp 85A specific band is present in MCMV85A infected cells. (C) Virus titre. Mice were infected with $10^6$ PFU eMCMV or MCMV85A and 6 days later MCMV titres were determined in salivary gland, liver and spleen. Each symbol represents one mouse. Empty circles indicate eMCMV and filled MCMV85A. Representative of two experiments.

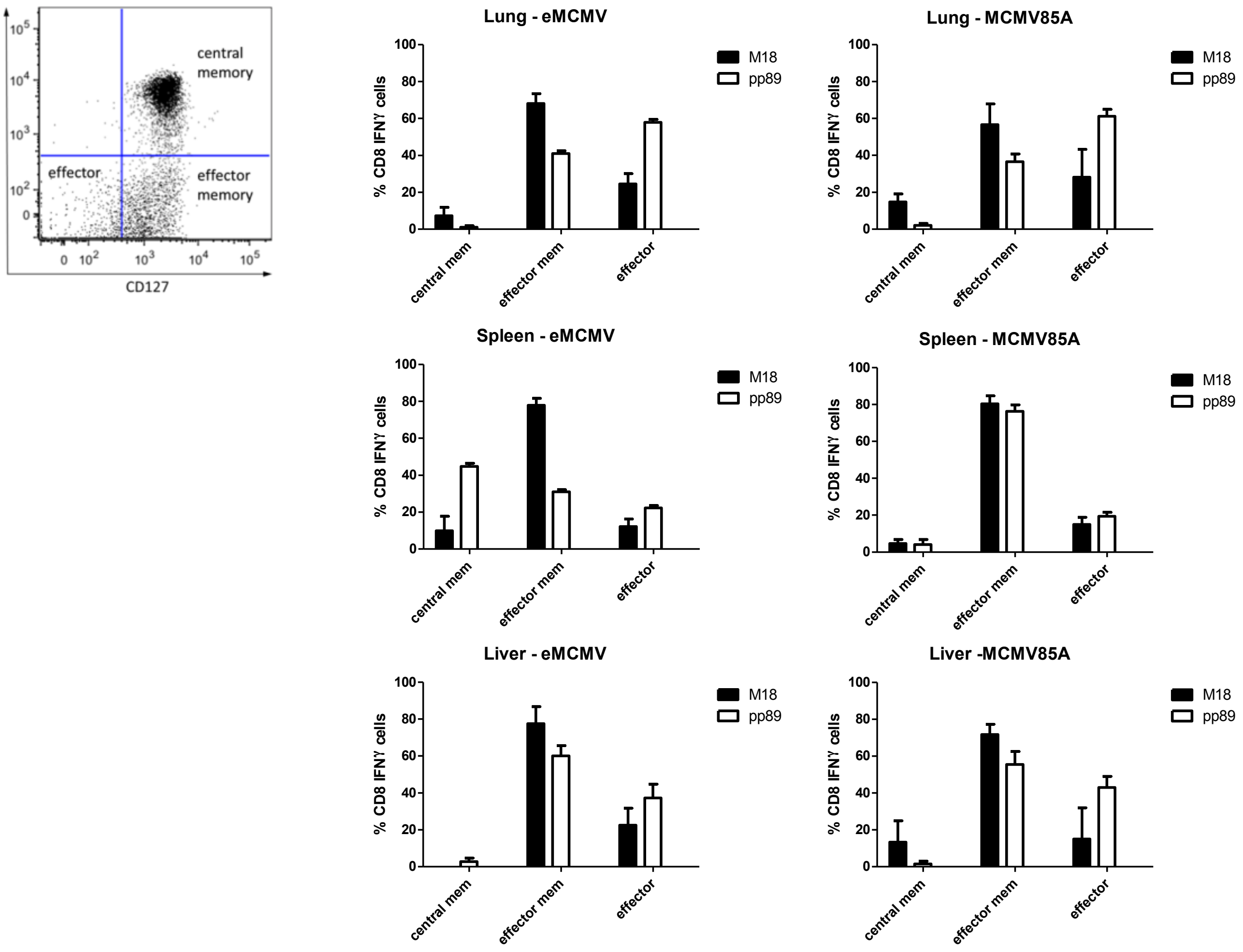


**Figure S2. Memory T cell subsets of pp89 and M18 specific cells in eMCMV or MCMV85A infected mice at d60**

BALB/c mice were infected with 2 x $10^6$ PFU eMCMV or MCMV85A I.P.. and sacrificed 60 days post infection. The proportion of pp89 or M18 central (CD62L+ CD127+), effector memory (CD62L- CD127+) or effector (CD62L- CD127-) antigen specific CD8 T cells in lungs, liver and spleen of each mouse, was determined by surface and intracellular immunofluorescence staining and flow cytometric analysis. The gates used are shown in the FACS plot. Results are expressed as the proportion of CD8 IFNγ cells with each phenotype and show the mean and SD of 3 mice.

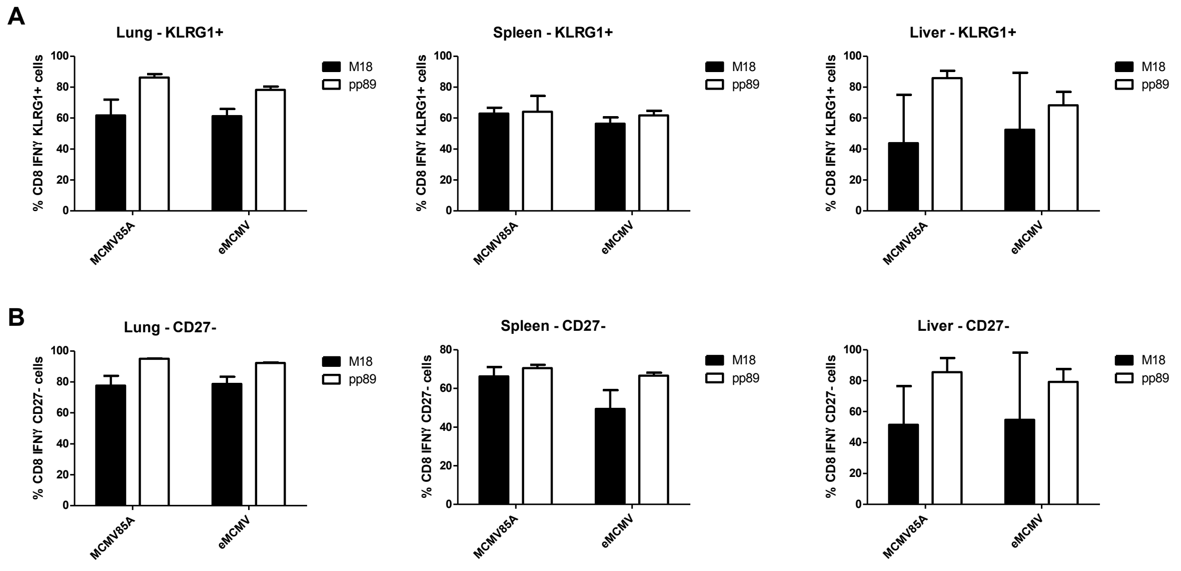


**Figure S3. Activation status of pp89 and M18 specific cells in eMCMV and MCMV85A infected mice.** BALB/c mice were infected with 2 x $10^6$ PFU eMCMV or MCMV85A I.P. and sacrificed 60 days post infection. The proportion of pp89 or M18 Mice were sacrificed 60 days post infection with eMCMV of pp89 or M18 antigen specific CD8 T cells was determined by surface and intracellular immunofluorescence staining and flow cytometric analysis after 6 hours stimulation of lung, spleen or liver cells with the pp89 or M18 peptides. Results are expressed as the proportion of pp89 or M18 specific CD8+ IFNg+ KLRG1+ (A) or CD8+ IFNg+ CD27- cells (B). Cells from 3 mice were assayed separately. Error bars show SD.

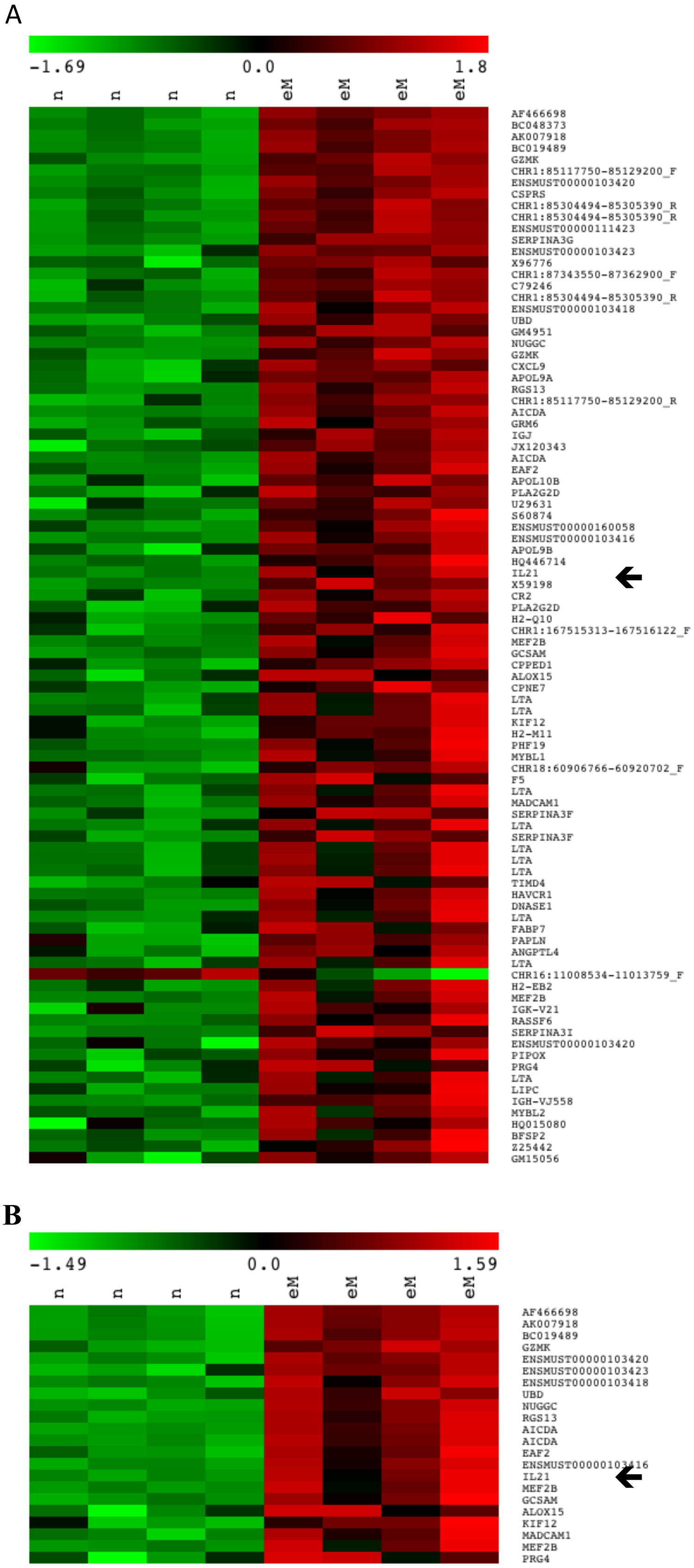


**Figure S4. Microarray heat maps.**

A. The heat map shows the top 100 most significant genes that are 2 fold up- or down-regulated, ranked by p value and expression level, mean centralized and divided by standard deviation (by row) (Z-score scaling). B. The top 22 most significant 3 fold up- or down-regulated genes are shown. Arrows indicate IL-21. Lung mononuclear cells of 4 naive (n) and 4 eMCMV infected mice (eM) were analyzed.
